# A Proteomic Approach Identifies Candidate Early Biomarkers to Predict Severe Dengue in Children

**DOI:** 10.1371/journal.pntd.0004435

**Published:** 2016-02-19

**Authors:** Dang My Nhi, Nguyen Tien Huy, Kaname Ohyama, Daisuke Kimura, Nguyen Thi Phuong Lan, Leo Uchida, Nguyen Van Thuong, Cao Thi My Nhon, Le Hong Phuc, Nguyen Thi Mai, Shusaku Mizukami, Lam Quoc Bao, Nguyen Ngoc Doan, Nguyen Van Thanh Binh, Luong Chan Quang, Juntra Karbwang, Katsuyuki Yui, Kouichi Morita, Vu Thi Que Huong, Kenji Hirayama

**Affiliations:** 1 Department of Immunogenetics, Institute of Tropical Medicine (NEKKEN), and Graduate School of Biomedical Sciences, Nagasaki University, Nagasaki, Japan; 2 Department of Clinical Product Development, Institute of Tropical Medicine (NEKKEN), and Graduate School of Biomedical Sciences, Nagasaki University, Nagasaki, Japan; 3 Department of Environmental and Pharmaceutical Sciences, Graduate School of Biomedical Sciences, Nagasaki University, Nagasaki, Japan; 4 Nagasaki University Research Centre for Genomic Instability and Carcinogenesis (NRGIC), Nagasaki, Japan; 5 Department of Molecular Microbiology and Immunology, Graduate School of Biomedical Sciences, Nagasaki University, Nagasaki, Japan; 6 Department of Immunology and Microbiology, Pasteur Institute, Ho Chi Minh City, Vietnam; 7 Department of Virology, Institute of Tropical Medicine (NEKKEN), and Graduate School of Biomedical Sciences, Nagasaki University, Nagasaki, Japan; 8 Nguyen Dinh Chieu Hospital, Ben Tre Province, Vietnam; Pediatric Dengue Vaccine Initiative, UNITED STATES

## Abstract

**Background:**

Severe dengue with severe plasma leakage (SD-SPL) is the most frequent of dengue severe form. Plasma biomarkers for early predictive diagnosis of SD-SPL are required in the primary clinics for the prevention of dengue death.

**Methodology:**

Among 63 confirmed dengue pediatric patients recruited, hospital based longitudinal study detected six SD-SPL and ten dengue with warning sign (DWS). To identify the specific proteins increased or decreased in the SD-SPL plasma obtained 6–48 hours before the shock compared with the DWS, the isobaric tags for relative and absolute quantification (iTRAQ) technology was performed using four patients each group. Validation was undertaken in 6 SD-SPL and 10 DWS patients.

**Principal findings:**

Nineteen plasma proteins exhibited significantly different relative concentrations (p<0.05), with five over-expressed and fourteen under-expressed in SD-SPL compared with DWS. The individual protein was classified to either blood coagulation, vascular regulation, cellular transport-related processes or immune response. The immunoblot quantification showed angiotensinogen and antithrombin III significantly increased in SD-SPL whole plasma of early stage compared with DWS subjects. Even using this small number of samples, antithrombin III predicted SD-SPL before shock occurrence with accuracy.

**Conclusion:**

Proteins identified here may serve as candidate predictive markers to diagnose SD-SPL for timely clinical management. Since the number of subjects are small, so further studies are needed to confirm all these biomarkers.

## Author Summary

Death outcome of dengue infection were mainly involved in pediatric patients with severe plasma leakage, or shock. No biomarker was addressed in the early stage of disease course for timely clinical management. Nineteen differentially expressed proteins were significantly detected in severe dengue patients with severe plasma leakage prior to the time of circulatory collapse occurrence. Angiotensinogen and antithrombin III level were verified to be significantly increased in early stage plasma of shock patients.

## Introduction

The last five decades saw a dramatic geographic expansion to 119 countries with overall 30 times more incidence of dengue infection, a mosquito-borne viral disease [1, 2]. The World Health Organization (WHO) estimates a mortality rate of dengue infection between 1 and 5%, with approximately 22,000 deaths, mainly among children with shock [2–4]. Most dengue infection are asymptomatic or self-limiting symptomatic while some patients, between days 4 and 7 of illness [5], may progress to severe disease, manifested with shock, serosal effusions and bleeding due to an increase of systematic vascular leak [1, 5]. The 2009 WHO guidelines, classifying dengue as dengue without warning sign (D), dengue with warning sign (DWS) and severe dengue [6], facilitate the clinical application and provide useful research endpoints [7]. SD contains any of severe plasma leakage leading to shock and/or respiratory distress (SD-SPL); severe bleeding, and severe organ impairment [1]. Prompt replacement of the circulating plasma losses can reduced the mortality rate to less than 1% among severe cases [1]. Early detection of the individuals who will develop shock among DWS patients is thus critical for timely clinical management. However, there is no objective biomarker in the early stage that reflects the probability of developing SD-SPL. Previously, albumin and transferrin levels were found to be significantly reduced in plasma at presentation day of shock compared with convalescent values, and urinary heparan sulfate and creatinine excretion was shown significantly greater in shock children compared with that in healthy control subjects [8]. In our recent study [9], circulating plasma DNA was found significantly higher in shock patients compared to non-shock on day 3 and 4 after the onset. In addition, serum hyaluronan level was also reported to be significantly higher in patients with shock from day 3 to day 7, compared to healthy subjects [10] while decreased plasma level of inter-α inhibitor proteins was shown to be correlated with shock at the time of hospitalization, but specific day of infection course during sampling was not notified [11]. However, these markers were investigated at the time of shock occurrence or a combination of times during the febrile phase and defervescence of the disease. It is, thus, difficult for clinicians to make an accurate prediction of SD-SPL before shock development.

“Warning signs” was mainly originated in the multicenter prospective study of more than 2000 dengue patients in seven countries in Asia and Latin American. However, not all DWS subjects progressed to severe dengue, just only 5% developed severe disease [12]. In an attempt to address helpful biomarkers for determining DWS patients at risk of SD-SPL, we conducted an observational study, using proteomic approach, from the hospital cohort called DENIM in Vietnam. Dengue patients were prospectively recruited and followed-up from febrile onset to defervescence as well as convalescent phase. The timing of early plasma sampling was carefully recorded and daily blood collection was carried out. In this study, we report for the first time the differential characterization of plasma proteome profiles and the identification of biomarkers for SD-SPL prediction among DWS children in the febrile phase, obtained from the DENIM cohort.

## Methods

### Ethics statement

The study was approved by Institutional Review Board of Institute of Tropical Medicine (NEKKEN), Nagasaki University (No.11063072) and the Pasteur Institute in Ho Chi Minh City (PIHCM) (No.602/QD-Pas 27/12/10). DENIM study is a longitudinal study of dengue fever, taking place in Nguyen Dinh Chieu Hospital in Ben Tre province, Vietnam. Children with age between 5 and 16 years hospitalized with clinically suspected dengue fever were eligible for enrollment. Written informed consent was given by a parent or guardian.

### Study population and sample collection

As described details in the study protocol (S1 Fig), briefly, children presenting acute onset fever (≥38°C) for less than 72 hours were enrolled. No patients had severe symptoms before hospitalization because of concerns about the confounding of previous treatment. At study entry, demographic data, history, and examination findings were recorded, and venous blood were obtained for initial dengue diagnosis. After hospitalization, the patients were diagnosed using standardized diagnostic techniques, as described below. Venous blood samples were daily collected at the time of admission, one day, two days and three days after hospitalization, at the time of shock, in the convalescent phase prior to discharge and two weeks after discharge from the hospital. Plasma was separated by centrifugation at 3000 rpm for 10 min and then divided into two tubes: one used for dengue verification and the remaining one frozen at −80°C for storage.

Routine laboratory tests includes complete blood counts, liver and renal function tests, ionograms were performed at the hospital laboratory. Hematocrit was measured every 8 hours in the day 1 and 2 from onset fever and every 4–6 hours in the day 3-day 6 from onset fever. During the time of severe dengue occurrence, hematocrit was measured every 0.5–1 hour (as shown in S1 Fig). On the day of defervescent, a right lateral decubitus chest X-ray was obtained once and could be repeated thereafter if the patients had symptoms of dyspnea or signs of pleural effusion in clinical examination as dullness to percussion, decreased tactile fremitus and asymmetrical chest expansion, reduced or inaudible breath sounds, egophony, or a friction rub. Echography was performed once or twice, especially when patients had abdominal pain or abnormal physical findings as above.

### Definitions

#### Dengue confirmed infection

Dengue infection was confirmed by positive serologic assays, virus isolation or RT-PCR for the identification of viral serotype, as described elsewhere [13]. Samples were considered serological positive for IgM and IgG if the ratio of optical density (OD) of test sera to OD of negative control was ≥2.3 [14]. IgM/IgG capture ELISA in paired acute and convalescent sera test were conducted to identify for primary and secondary dengue infection. The case was diagnosed as secondary infection if the DV IgM/IgG ratio was <1.8 [6]. Viral RNA was also extracted for the molecular detection of Dengue virus and conformation of its serotype [15]. The serotype was then determined by semi-nested PCR using specific primer sets to amplify serotype-specific fragments from the regions encoding the capsid and membrane proteins of Dengue virus [16].

#### Classification of severe dengue and dengue warning sign

Patients were classified according to 2009 WHO criteria [1], see details in Table 1. For all patients, the earliest plasma samples collected at 6–48 hours prior to the development of SD-SPL or defervescence were included in the study. The window of 6–48 hours is long enough to measure the biomarkers before the progress of SD-SPL for clinical practice.

**Table 1:** Dengue case classification. An acute febrile patient with a confirmed diagnosis of dengue virus infection was considered as Dengue with warning sign (DWS) or SD-SPL modified from the criteria of 2009 WHO guidelines.

Dengue with warning sign (DWS)	Severe dengue [6]
Abdominal pain	Severe plasma leakage leading to shock (weak and rapid pulse, narrow pulse pressure ≤20 mmHg, or hypotension for age with cold, clammy extremities) (SD-SPL)
Persistent vomiting	Severe bleeding
Clinical fluid accumulation	Severe organ impairment (acute liver failure: AST or ALT ≥1000 UI/L, impaired consciousness, cardiomyopathy and other organ)
Mucosal bleeding	
Lethargy or restlessness	
Liver enlargement >2 cm	
Increase in hematocrit (Hct) with rapid decrease in platelet count	

#### Isobaric tags for relative and absolute quantification (iTRAQ) labeling and mass spectrometry analyses

Individual iTRAQ labeling was suggested to observe the variation among eight subjects while highlighting the most consistent disease-specific changes (S2 Fig). For 8-plex analyses, four DWS and four SD-SPL samples, randomly selected from two groups was individually used. Following immune-depletion of high-abundance proteins using immobilized specific IgY 14 Spin columns kit (Seppro, Sigma Aldrich), each of depleted plasma was centrifugally concentrated and buffer exchanged. The concentration of plasma protein was determined using BCA assays (Pierce Thermo, US). Then, 100 μg of protein from each individual plasma in Tri-ethyl-ammonium-bicarbonate/0.1% SDS (AB Sciex, US) was reduced with 50 mM Tris-(2-carboxylethyl) phosphine (AB Sciex, US), alkylated with 200 mM methyl methane thio-sulfonate (AB Sciex, US), digested with trypsin CaCl_2_ (AB Sciex, US) at a ratio 1:5 (w/w trypsin: sample), and labeled with one of the isobaric reagents as described in the S1 Appendix. Each digested sample of four DWS subjects were labeled with iTRAQ reagent 113, 114, 115, 116, respectively and those of four SD-SPL individuals were allocated to the tags 117, 118, 119, and 121. The resulting labeled peptide samples were then pooled together, dried up and then, the mixtures were fractionated by strong cation exchange separation and analyzed by mass spectrometry as described in the S1 Appendix.

### Data analysis

Spectra acquired from the iTRAQ experiment were submitted to the ProteinPilot Software (version 4.0, AB Sciex), using Paragon protein database search algorithm [17], for generation of peak list, protein identification and quantification. Protein ontology classification was performed using PANTHER classification system (http://pantherdb.org/, CA).

### Western blotting

Angiotensinogen and antithrombin III were selected for validation in sixteen subjects individually (ten DWS and six SD-SPL). Western blotting was performed after SDS-PAGE with primary antibodies: anti-AGT (Angiotensinogen, Abcam) and anti-AT III (Antithrombin-III, GeneTex Inc). For specific details, see S1 Appendix.

### Statistical analysis

All statistical analyses were performed using SPSS version 17.0 and MedCalc version 14.12. Statistical significance was determined by non-parametric Mann Whitney *U-*test for iTRAQ data comparison and validation data analysis. Experimental data of WB analysis were presented as median ± interquartile range. χ^2^ was used for categorical analysis, Fisher’s exact test was used when the expected counts were less than 5. A comparison of different methods for the selected feature was determined by the area under the curve (AUC) of receiver operating characteristics (ROC). An AUC value >0.75 was used as a threshold for good diagnostic test [18]. The DeLong test was performed to compare AUCs. A multivariate logistic regression model was used to observe the independent predictive value of the biomarker on disease outcome. The difference was considered significant at p<0.05.

### Accession numbers

sp|P01019|ANGT_HUMAN, sp|Q9UFD9|RIM3A_HUMAN, **s**p|Q9Y4P8|WIPI2_HUMAN, **s**p|P01008|ANT3_HUMAN, sp|Q8WZ42|TITIN_HUMAN, sp|P00450|CERU_HUMAN, sp|P02765|FETUA_HUMAN, sp|P02787|TRFE_HUMAN, sp|Q68DK2|ZFY26_HUMAN, sp|P00734|THRB_HUMAN, sp|Q8WXH0|SYNE2_HUMAN, sp|O95255|MRP6_HUMAN, sp|Q9NUV7|SPTC3_HUMAN, sp|Q8TEM1|PO210_HUMAN, sp|Q8NGK0|O51G2_HUMAN, sp|Q7RTS5|OTOP3_HUMAN, sp|Q15582|BGH3_HUMAN, sp|Q8WWN8|ARAP3_HUMAN, sp|Q5THJ4|VP13D_HUMAN

## Results

### Characteristics of the study subjects

**Fig 1:**
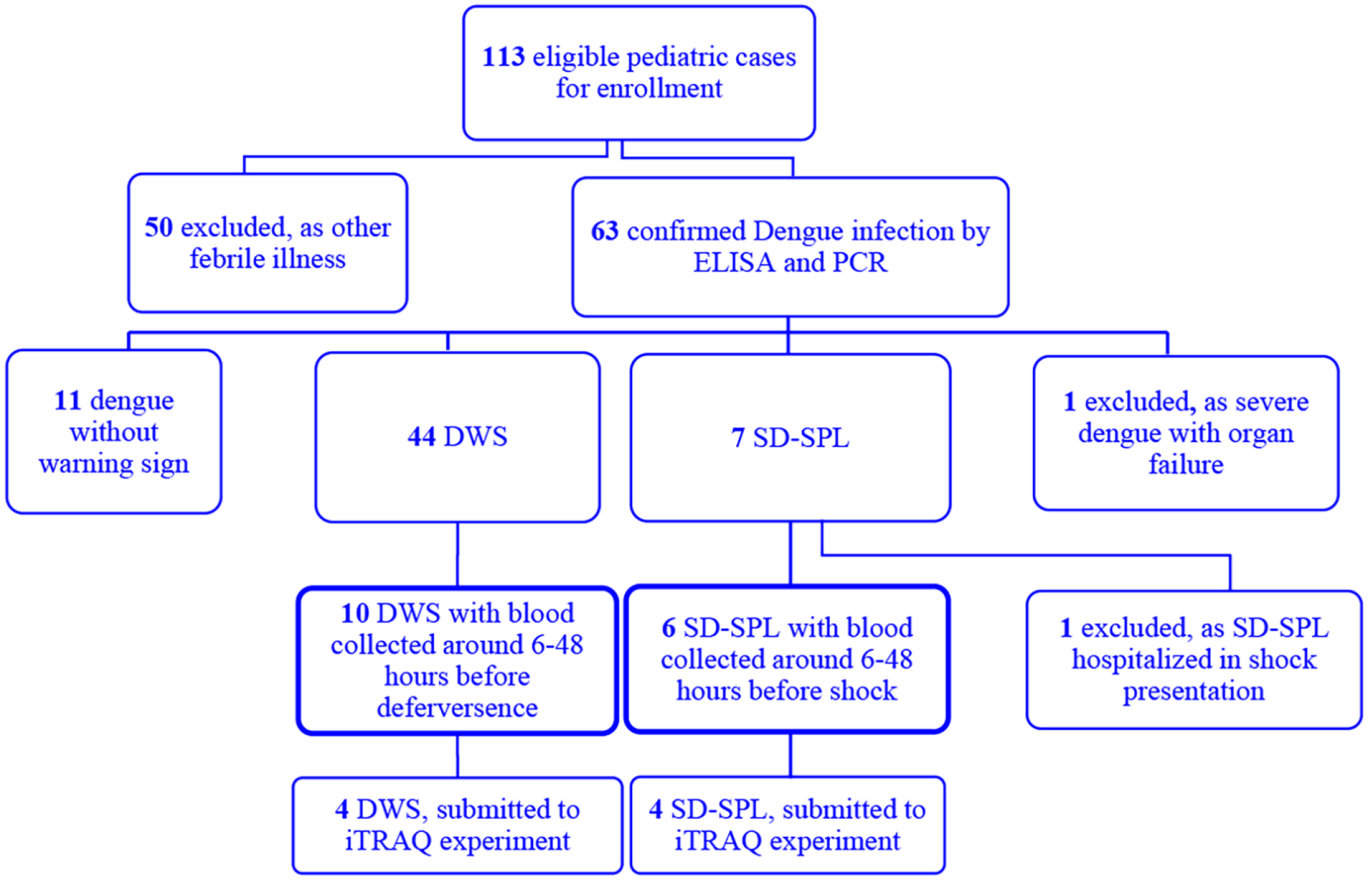
Study profile. Of 113 patients enrolled, 63 had laboratory-confirmed dengue infection. One patient hospitalized in shock presentation was excluded. Six SD-SPL and ten DWS, with early stage plasma collected, were included in the current study.

A flow chart of recruitment in the prospective DENIM study is presented in Fig 1. From January 2011 through June 2012, 113 children were enrolled and 63 had confirmed dengue infection. After classification, 44 patients with diagnosis of dengue fever with warning sign without shock presentation (DWS) and seven patients with diagnosis of severe dengue with severe plasma leakage (SD-SPL) underwent study selection. One of SD-SPL was excluded due to the shock occurrence at the time of hospitalization. All of six cases of SD-SPL had clinical shock presentation with narrow pulse pressure, quick pulse rates (as in S2 Table). No patients showed fluid accumulation without or with respiratory distress and no abnormality in echography and chest X-ray were recorded at the time of shock occurrence. One case had fluid overload with pleural effusion after intravenous fluid therapy and needed treatment of small doses of diuretic (S2 Table). In this study, for addressing factors that can provide the early prediction of shock development in preceding 6–48 hours, a sub-set of 16 of the pediatric dengue patients (ten DWS and six SD-SPL) with pre-defervescence or pre-shock plasma were included. Patients selected in DWS and SD-SPL groups showed similar age and gender distribution (Table 2). No significant differences of maximal hematocrit (Hct) and minimal platelet count during the first three day from fever onset were found between the two groups. Hct and platelet count obviously changed from day 4 or 5 of disease course in all subjects, only one case of SD-SPL had maximal Hct on day 3. There was no difference of maximal hematocrit during disease course between two groups and the median day of maximal Hct was day 4 from onset fever for both of two groups. Hematocrit features and fluid management of sixteen patients were shown in S1 Table. The percentage of Hct rising of individual subjects was also recorded (S1 Table). Dengue serologic titration indicated that secondary infection was most common in both DWS (70%) and SD-SPL groups (100%). No difference was recorded in timing of sampling between DWS and SD-SPL subjects. The median duration of sampling time was 28.5 hours prior to shock presentation for SD-SPL group and 28 hours before defervescence for DWS controls (Table 2).

**Table 2:** Characteristics of dengue patients.

	DWS (n = 10)	SD-SPL (n = 6)	p^a^
Demographic features			
Age—median year, IQR	9 (8–11.5)	10 (8.5–12.7)	0.4
Gender (F:M)	6:4	4:2	1
Clinical symptoms and physical signs			
Abdominal pain	5 (50%)	3 (50%)	
Persistent vomiting	2 (20%)	4 (67%)	
Mucosal bleeding	2(20%)	2 (33%)	0.6
	1 vaginal (D5)	1 vagina (D4)	
	1 GI, minor (D3)	1 nasal (D6)	
Liver enlargement > 2cm	0 (0%)	1 (17%)	
Narrow pulse pressure	0	6	
Shock manifestation	0	6	
Laboratory tests			
Hct max until day 3 –% median, IQR	43 (39.4–47.7)	43.5 (37.7–47.7)	0.9
Platelet min until day 3 (× 103/μl)–median, IQR	141.5 (74.7–197.3)	113.5 (55.7–155)	0.4
Hct max during disease course–% median, IQR	45.2 (40.2–47.7)	47 (46.2–50.3)	0.1
Platelet min during disease course (× 103/μl)–median, IQR	87.5 (60–127)	41 (30.3–102.3)	0.09
Day of Hct max—median day, range	4 (4–6)	4 (3–6)	1
Day of platelet min—median day, range	5 (4–7)	4.5 (3–6)	0.5
PI/SI	3/7 (30%/70%)	0/6 (100%)	
Virus serotype—DENV 1/2/3/4	3/2/2/2	0/3/0/1	
Day of sampling from onset fever—median day, IQR	3 (3–3.3)	3 (2.8–4.3)	0.75
Time of sampling prior to shock—median hour, IQR		28.5 (10.5–40.3)	0.7
Time of sampling prior to defervescence—median hour, IQR	28 (20.8–44.3)		

Hct max, maximal hematocrit; PI/SI, primary/secondary infection; IQR, interquartile range; GI, gastrointestinal;

D, day from onset fever;

^a^For categorical variables, χ^2^ test or Fisher’s exact test (F). For continuous variables, non-parametric Mann-Whitney *U* test

### Plasma iTRAQ analysis for identification of candidate biomarkers of SD-SPL

For discovery of potential markers of SD-SPL, we used eight plasma samples in the early stage of dengue infection including four SD-SPL and four DWS samples as controls, randomly selected from two groups of sixteen subjects. The plasma level of high abundant proteins was determined by BCA assays before high-abundance protein depletion (as shown in S3 Fig). No significant difference of the concentration of plasma protein between two patient groups was observed. As shown in S3 Table, the relative ratios for remaining amounts of abundant proteins (including albumin, immunoglobulin, α1-antitrypsin, transferrin, haptoglobin, α2-macroglobulin, fibrinogen, α1-acid Glycoprotein (orosomucoid), apolipoproteins A-I) between two groups of patients showed no difference, except for transferrin.

**Fig 2:**
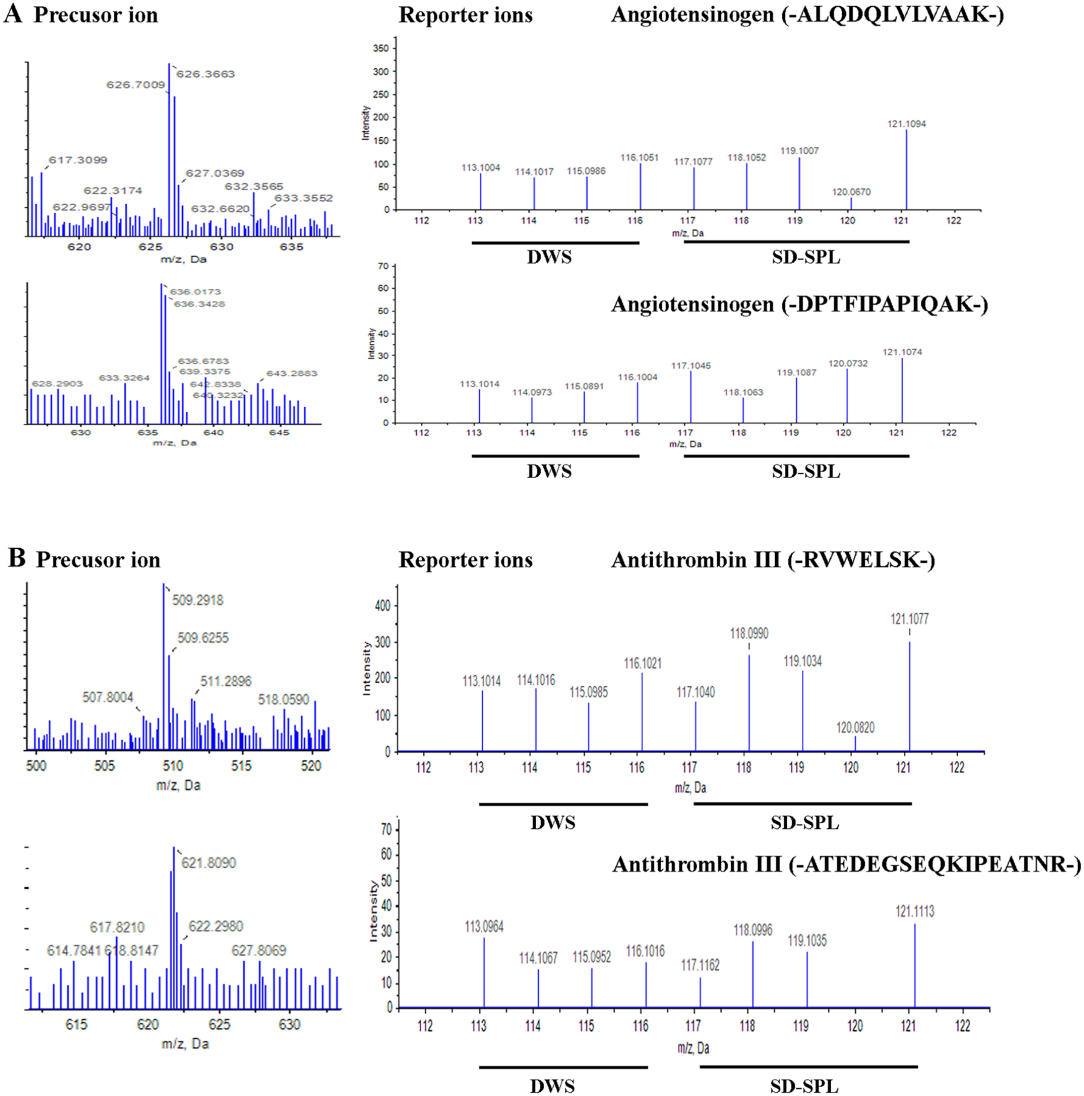
Identification of angiotensinogen (AGT) and antithrombin III (AT III) proteins. Representative precursor ion and MS/MS spectra of reporter ions from peptide ALQDQLVLVAAK and DPTFIPAPIQAK of AGT (A) and from peptide RVWELSK and ATEDEGSEQKIPEATNR of AT III (B). Quantification was derived from the signal intensities of eight iTRAQ reporter ions.

Seventy proteins were identified and quantified with a confidence level of 95% from iTRAQ experiment (S3 Table). Fig 2 shows representative MS and peptide MS/MS spectrum of the corresponding amino acid sequences used in the identification and quantitation of angiotensinogen (AGT) and antithrombin III (AT-III) proteins in this study. Peaks of unique peptide sequence -ALQDQLVLVAAK- and -DPTFIPAPIQAK- corresponding to AGT protein, and -RVWELSK- and -ATEDEGSEQKIPEATNR-, corresponding to AT III proteins (Fig 2), revealed the increased signal intensities in the SD-SPL plasma. The relative intensities of iTRAQ reporter ions used to quantify the relative expression of these proteins were also shown in Fig 2.

A statistical comparison revealed that 19 human proteins in both SD-SPL and DWS groups were significantly differentially expressed (p<0.05), with 5 over-expressed and 14 under-expressed relative to DWS patients (Table 3). The fold-change of each plasma protein level between DWS and SD-SPL patients was also determined (Table 3) with the maximal and minimum ratios, 1.2 and 2.4, respectively. The proteins were found to participate in various biological processes including blood coagulation, vascular regulation, cellular and transport-related processes and immune response with expected function of protease, protease inhibitor, transaminase, membrane traffic/transfer/carrier proteins, cell adhesion molecule and signaling molecule as shown in Table 4.

**Table 3:** List of differentially expressed plasma proteins of SD-SPL compared with those of DWS (p<0.05).

Accession No^a^	Description	p-value	Fold change (relative to DWS patients)
**Over-expressed**			
sp|P01019|ANGT_HUMAN	Angiotensinogen	0.0117	1.2
sp|Q9UFD9|RIM3A_HUMAN	RIMS-binding protein 3A	0.0426	1.8
sp|Q9Y4P8|WIPI2_HUMAN	WD repeat domain Phosphoinositide-interacting protein 2	< 0.0001	1.5
sp|P01008|ANT3_HUMAN	Antithrombin III	0.0031	1.4
sp|Q8WZ42|TITIN_HUMAN	Titin	0.0272	1.3
**Under-expressed**			
sp|P00450|CERU_HUMAN	Ceruloplasmin	0.0332	1.2
sp|P02765|FETUA_HUMAN	Alpha-2-HS-glycoprotein	0.001	1.3
sp|P02787|TRFE_HUMAN	Serotransferrin	0.0083	1.3
sp|Q68DK2|ZFY26_HUMAN	Zinc finger FYVE domain-containing protein 26	0.0248	1.6
sp|P00734|THRB_HUMAN	Prothrombin	0.0203	1.3
sp|Q8WXH0|SYNE2_HUMAN	Nesprin-2	0.0184	1.3
sp|O95255|MRP6_HUMAN	Multidrug resistance-associated protein 6	0.0021	1.2
sp|Q9NUV7|SPTC3_HUMAN	Serine palmitoyltransferase 3	0.0078	2.3
sp|Q8TEM1|PO210_HUMAN	Nuclear pore membrane glycoprotein 210	0.0135	2.4
sp|Q8NGK0|O51G2_HUMAN	Olfactory receptor 51G2	0.001	1.5
sp|Q7RTS5|OTOP3_HUMAN	Otopetrin-3	0.015	2.3
sp|Q15582|BGH3_HUMAN	Transforming growth factor-beta-induced protein ig-h3	0.0024	2.2
sp|Q8WWN8|ARAP3_HUMAN	Arf-GAP with Rho-GAP domain, ANK repeat and PH domain-containing protein 3	0.0248	1.4
sp|Q5THJ4|VP13D_HUMAN	Vacuolar protein sorting-associated protein 13D	0.0109	1.5

^a^Swissprot database (sp)

**Table 4:** Biological analysis of identified proteins using PANTHER classification.

Name	Protein class & Biological process	Pathway
Angiotensinogen	Serine protease inhibitor	Vascular regulation
Rims-binding protein 3A	Cellular process	
WD repeat domain phosphoinositide-interacting protein 2	Regulate autophagy	
Antithrombin III	Serine protease inhibitor	Blood coagulation
Titin	Cell adhesion molecule	
Ceruloplasmin	Extracellular matrix	Membrane-bound signaling molecule
Alpha-2-hs-glycoprotein	Cysteine protease inhibitor	
Serotransferrin	Transfer/carrier protein	
Zinc finger FYVE domain-containing protein 26	Intracellular protein transport, Membrane traffic protein	
Prothrombin	Serine protease	Blood coagulation
Nesprin-2	Non-motor actin binding protein	
Multidrug resistance-associated protein 6	ATP-binding cassette (abc) transporter	
Serine palmitoyltransferase 3	Transaminase	
Nuclear pore membrane glycoprotein 210	Membrane traffic protein	
Olfactory receptor 51g2	G protein-coupled receptor	
Otopetrin-3	Membrane traffic protein	
TGF beta-induced protein ig-h3	Signaling molecule; cell adhesion molecule	
Arf-gap with Rho-gap domain, ANK repeat and PH domain-containing protein 3	Nucleic acid binding; g-protein modulator	
Vacuolar protein sorting-associated protein 13d	Involved in trafficking of membrane proteins	

Proteins were categorized to one or more profiles of both protein class, biological processes and pathway based on gene ontology.

### Validation of candidate markers in individual plasma samples

**Fig 3:**
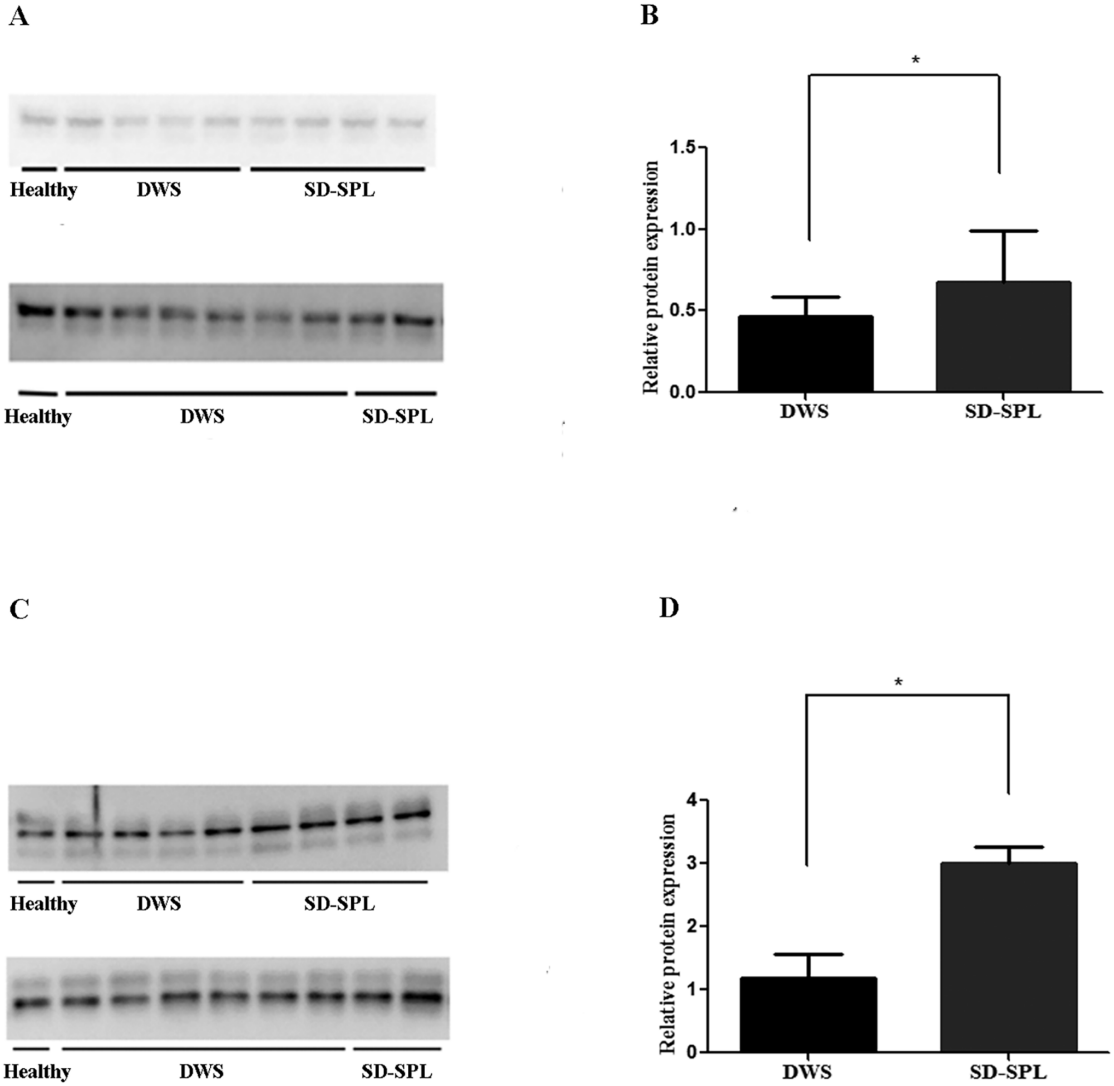
Western-blot analysis validating iTRAQs results for angiotensinogen and antithrombin III. Representative protein bands in DWS (n = 10) and SD-SPL (n = 6) for AGT (A) and for AT III (C). Quantification of relative protein expression of AGT (B) and AT III (D), based on normalized densitometry to healthy control, *p < 0.05.

Based on the functional properties of these AGT and AT III (Table 3) we selected those for further study. Verification of those two proteins detected by iTRAQ method was performed by Western blotting in 16 individual plasma samples (10 DWS and 6 SD-SPL). Relative quantitation of AGT (Fig 3A and 3B) and AT III (Fig 3C and 3D) blots indicated that the levels of these proteins were significantly higher in early stage plasma of SD-SPL patients (n = 6) compared to those of the control group (n = 10) (p = 0.031 for AGT and p = 0.017 for AT III).

### Assessment of probability to predict SD-SPL of candidate markers

**Fig 4:**
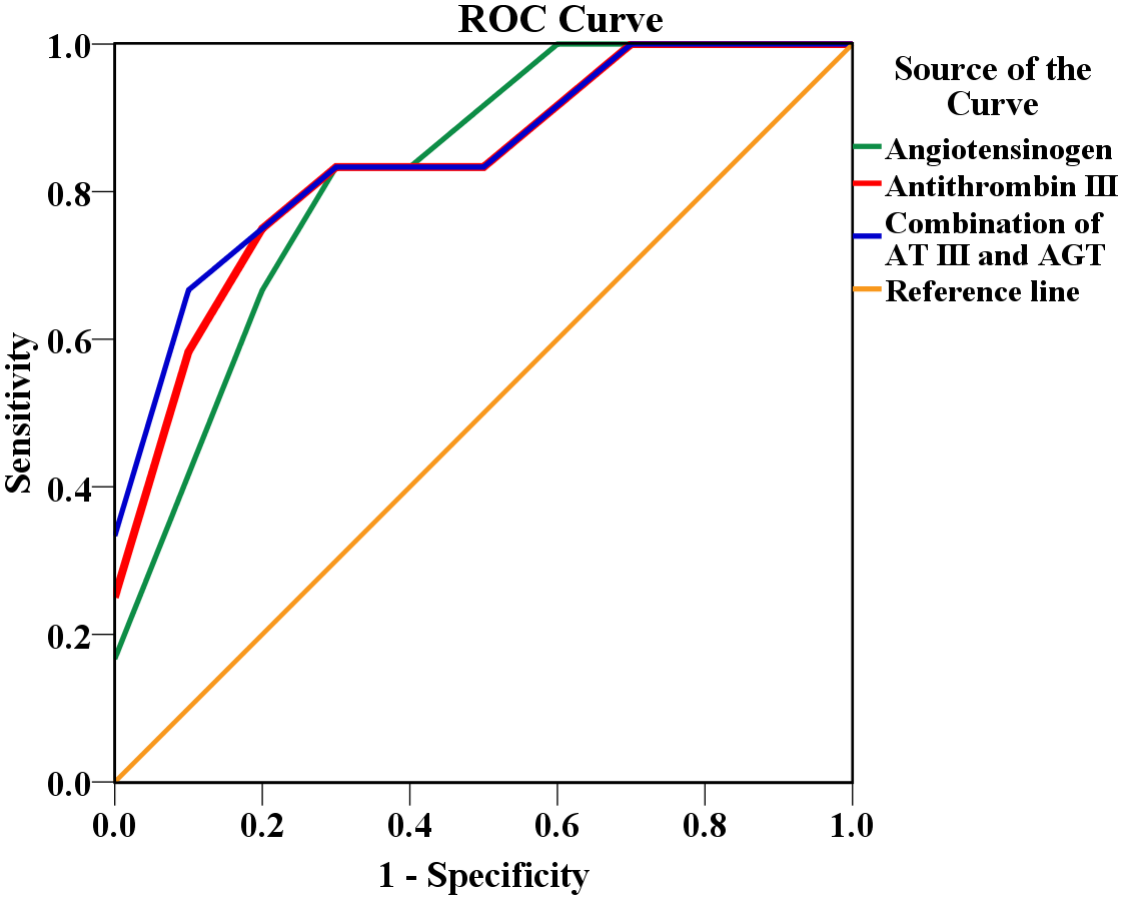
Receiver operating characteristic (ROC) curves comparing antithrombin III and angiotensinogen markers for SD-SPL prediction. Area under the curve (AUC) for antithrombin III = 0.85, AUC for angiotensinogen = 0.83 and AUC for combination of two markers = 0.87.

The univariate logistic regression analysis revealed that level of the relative expression of AT III as well as AGT in plasma significantly correlated with the risk of SD-SPL (odds ratio (OR) 5.93, 95% confidence interval (CI) 1.23–28.68, p = 0.02; and OR 20.00, 95% CI 1.42–284.45, p = 0.03, respectively). ROC curve analysis indicated that SD-SPL prediction by AT III candidate performed more accurately with area under the curve (AUC) of 0.85±0.11 (p = 0.0009) compared to AGT marker (AUC = 0.83±0.10, p = 0.0013) although no statistically significant difference between the two curves was observed (Fig 4). Combination of AGT and AT III achieved an AUC of 0.87±0.10 (p = 0.0004), however, there was no significant difference between this curve and AGT and AT III curves on their own (Fig 4). AT III has the potential to be a more precise marker for shock anticipation. Thus, the relative plasma level of AT III together with other variables (age, maximal hematocrit and minimal platelet count) recorded during the first three day from the onset fever were included in multivariate logistic regression analysis. The analysis demonstrated that the relative concentration of AT III in plasma was independently (p = 0.03) associated with a greater risk of SD-SPL (Table 5).

**Table 5:** Multivariate logistic regression analysis of SD-SPL predictive diagnosis.

Factors	Univariate			Multivariate		
	OR	95% CI	p	OR	95% CI	p
Age	1.2	(0.78–1.83)	0.4	1.42	(0.53–3.78)	0.48
Hct max until day 3 (%)*	1.03	(0.85–1.25)	0.8	0.95	(0.62–1.48)	0.83
Platelet min until day 3 (× 10^3^/μl)*	0.99	(0.97–1.01)	0.3	0.98	(0.95–1.02)	0.28
Antithrombin III	5.93	(1.23–28.68)	0.02	6.52	(1.06–40.11)	0.03
Angiotensinogen	20.00	(1.42–282.45)	0.03			

OR, Odds ratio;

CI, Confidence interval

*odds ratio represents the incremental odds of SD-SPL prediction for every unit increase of one percent in Hct max or 1000 platelet per microliter in Platelet min

## Discussion

The severe dengue with severe plasma leakage (SD-SPL) is known as the most common of serious manifestation of severe dengue. Using iTRAQ and mass-spectrometry analysis, we have identified five proteins over-expressed and 14 proteins under-expressed in the early stage plasma of SD-SPL patients compared to that of dengue with warning signs (DWS) as shown in Table 3. Moreover, functionally selected two proteins from those, AGT and ATIII were confirmed by Western blotting analysis using more number of cases (Fig 3).

While there was not much difference in the fold-change between iTRAQ experiment and Western blotting analysis of AGT, AT-III had higher change in immunoblot analysis than iTRAQ approach. These differences could be attributed to the use of depleted or whole plasma, the small sample size, or inherent differences in experimental techniques. Although iTRAQs approach was demonstrated as a robust and reproducible mean for simultaneous identification and quantitation of all peptides, and its advantage over other labelings (i.e., ICAT, SILAC, DIGE, mTRAQ) have been reported previously [19, 20], underestimation of the change in iTRAQ analysis between samples has been recently proposed to depend on the MS-platform (QTOF, TOF, QTRAP) [21, 22]. In concordance with our result, orthogonal analysis, known as biochemical assays such as antibody-based Western blots or enzyme-linked immunoassays, have previously shown a fold-change that is higher than the corresponding ratios derived from iTRAQ approach [22].

Angiotensinogen (AGT) detected increased here has never been reported as a predictive marker of shock in Dengue infection before. It is known as the renin substrate, a precursor molecule of angiotensin I & II, and plays an important role in the renin-angiotensin system [23]. Changes in mRNA expression levels of endothelial-like cells following infection with dengue virus type 2 showed the up regulation of angiotensinogen [24] and the high plasma levels of angiotensin II impair endothelial cell (EC) function [25], inhibit EC motility [26] and induce apoptosis of human ECs [27–29].

Although both of AGT and AT III showed correlation with SD-SPL predictive diagnosis, AT III candidate was the more precisely performing marker at discriminating circulatory collapse from DWS patients (AUC 0.85). Through multivariate analysis, our study demonstrated that for prediction of SD-SPL in early time of disease course, AT III was independent of other features as hematocrit and platelet count, which have been usually used to monitor clinical progress. In the current study, they reveal obvious changes from day 4 of disease or around the time of shock occurrence, concordant with previous paper [5]. Besides, elevation of AT III accompanied with the low level of prothrombin in pre-shock plasma of SD-SPL patients may indicate the reduction of thrombin formation and that the clotting time for plasma is prolonged. That was consistent with the higher tendency of mucosal bleeding in SD-SPL group (20% of DWS versus 33.3% of SD-SPL) during disease course. Among our patients with a hemorrhagic tendency, mucosal hemorrhage was mostly observed from day 4 of the disease with minor bleeding. However, children who will subsequently develop SD-SPL did have significantly abnormal levels of circulating level of AT III and prothrombin during the first few days.

AT III has previously been found to have lower levels in the plasma of SD-SPL patients resuscitated from shock with colloid fluids compared with SD-SPL patients treated with electrolyte fluids [30]. Un-estimated dilution effect due to intravenously fluid perfusion before the sampling time may potentially explain the decrease of this coagulation inhibitor and the accuracy of that estimation is difficult to assess. In our study, plasma was collected before the patients had received fluids so the level of AT III in plasma of SD-SPL patients should reflect disease pathogenesis. Interestingly, AT III has also been shown to prevent the shedding of the endothelial glycocalyx which can causes a substantial increase in vascular permeability [31] and platelet adhesion [32]. In the pre-shock period the increase of AT III may be a natural human response to minimize the relative damage of glycocalyx layer.

We have identified markers that could have a role in predicting severe dengue with severe plasma leakage among dengue patients with warning signs. Such biomarkers may help the institution of timely management and help guide dengue treatment. The study is limited by the small number of patients studied and this may a principle to apply the technique in further study with lager population for exploring the threshold of these markers in predictive diagnosis of severe dengue.

## Supporting Information

S1 Appendix Experiment procedures. (DOCX)

S1 Fig Flowchart of study process in hospital. (TIF)

S2 Fig Schematic presentation of experimental design showing biomarker identification by iTRAQ approach combined with nLC-ESI-MS/MS. (TIF)

S3 Fig Determination of protein content in plasma before removals of high abundant proteins. Bar chart represents the median of protein concentration with the upper error bars indicate the 75th percentile of inter-quartile range.
(TIF)

S1 Table Classification, sampling time, hematocrit features and fluid management of sixteen patients. (DOCX)

S2 Table Features of SD-SPL patients at the time of shock occurrence. (DOCX)

S3 Table Proteins identified and quantified in iTRAQ experiment. (DOCX)
