# Supplementary material for: A Proteomic Approach Identifies Candidate Early Biomarkers to Predict Severe Dengue in Children
Source: PLoS Negl Trop Dis. 2016 Feb 19;10(2):e0004435. doi: 10.1371/journal.pntd.0004435 (PMC4764501; doi:10.1371/journal.pntd.0004435)
Supplement: S1 Appendix — (DOCX) [file pntd.0004435.s001.docx]

**S1 Appendix. Experiment procedures**

**Chemical reagents**. Acetonitrile, formic acid, ammonium formate, trifluoroacetic acid (TFA) and ultrapure HPLC grade water were purchased from Wako Pure Chemical Industries, Ltd.

**iTRAQ labeling of individual plasma.** Immune-depletion of highly-abundant proteins (HAPs) using immobilized specific IgY 14 Spin columns kit (Seppro, Sigma Aldrich) was applied to equal volume of individual plasma, according to the manufacturer’s instruction. Briefly, 0.45 µm-filtered plasma were diluted in dilution buffer (Tris Buffered Saline: 10 mM Tris-HCl, pH 7.4, 150 mM NaCl), washed, and equilibrated in the immobilized specific IgY 14 Spin column. Flow-through fractions containing low-abundance proteins were collected, concentrated by centrifugation and buffer exchanged. The concentration of plasma protein was determined using BCA assays (Pierce Thermo, US). The amount of plasma protein used in the experiments was adjusted to the total plasma protein of each patients. For 8-plex iTRAQ experiment, 100 μg of protein from each individual plasma in Tri-ethyl ammonium bicarbonate / 0.1% SDS (AB Sciex, US) was reduced with 50 mM Tris- (2-carboxylethyl) phosphine (AB Sciex, US) followed by incubation at 60°C for 1 hr. The reduced cysteine residues were blocked by incubation with alkylation of 1 μl of 200 mM methyl methane thio-sulfonate (AB Sciex, US) at room temperature for 15 min. The protein was then digested by the addition of trypsin CaCl_2_ (AB Sciex, USA) at a ratio 1:5 (w/w trypsin: sample) in 50 mM TEAB (Sigma Aldrich), and incubated at 37°C overnight. Tryptic digested peptides were desalted by C18 columns (Protea Biosciences, US). After being dried in a SpeedVac concentrator (Thermo Scientific, US), they were reconstituted in 33 μl of 0.5M TEAB (AB Sciex, US). One unit of each iTRAQ reagent label (AB Sciex, US) was thawed and reconstituted in 50 μl of isopropranolol (AB Sciex, US). The contents of each reagent solution was added to the digested peptides and incubated at room temperature for 2 hours. Digested sample of four DFWS subjects were labeled with iTRAQ reagent 113, 114, 115, 116, respectively and those of four DSS individuals were allocated to the four remaining tags 117, 118, 119, and 121. The resulting labeled peptide samples were then pooled together and dried up under vacuum centrifugation (SpeedVac concentrator, Thermo Scientific, US) before peptide fractionation.

**Peptide Fractionation with Strong Cation Exchange Chromatography.** After drying, labeled mixture was reconstituted in strong cation exchange (SCX) buffer (5mM ammonium formate in 20% acetonitrile, pH 3.0) and fractionated with SCX Spin Tips (Protea Biosciences, US), as described in manufacturer’s guide with minor modification. Briefly, the reconstituted sample solution was loaded onto the SCX column centrifugally. The non-absorbed flow-through was collected. Seventeen different solutions were used for step-wise peptide fractionation (20, 30, 40, 50, 60, 70, 80, 90, 100, 150, 200, 250, 300, 350, 400, 450, 500 mM ammonium formate in 10% acetonitrile). A total of 18 fractions were collected, dried under vacuum centrifugation (SpeedVac) and then submitted in nano liquid chromatography – electrospray ionization – time of flight mass spectrometer (nLC-ESI-TOF/TOF-MS/MS) for protein identification and quantitation.

**Mass Spectrometry Analyses with iTRAQ labeling.** All nLC-MS/MS (nano-liquid chromatography-tandem mass spectrometry) experiments were performed on a QSTAR-XL mass Spectrometer (AB Sciex) retrofitted to a nano-Advance UHPLC (AMR Inc) equipped with CTC PAL HTS autosampler (AMR Inc). The nLC-MS/MS system was controlled by processing and data acquisition software Analyst QS version 1.0 (AB Sciex). Individual dried SCX fractions were freshly reconstituted in 20 μl of 0.1% TFA in 2% acetonitrile. A volume of 2 μl of filtered sample solution was injected and then eluted onto a 0.1 × 150 mm reverse phase column Zaplous alpha Pep C18, 120 Å pore, 3.0 μm particle (AMR Inc) combined to the nano-electrospray source (AB Sciex) and connected to a 1P-4P coated PicoTip nano-electrospray emitter (SilicaTip^TM^_,_ New Objective, US). An online trap column (CERI) was connected between the pump outlet tubing and separation column to minimize sample chemical contaminants. Peptides were eluted into the MS/MS system at a constant flow rate of 500 nL/min with a gradient from 95% mobile phase A to 45% mobile phase B over 60 min (solvent A: 0.1% formic acid in 100% ultrapure water, solvent B: Acetonitrile), then 95 % mobile phase B in 5 min and an additional 10 min of 5 % mobile phase B was held at the end of each run. The total duration of an LC run was 75 min, including sample loading, peptide elution and column re-equilibration. The mass QSTAR XL system (AB Sciex) was operated in an information-dependent acquisition (IDA) mode using Analyst QS version 1.1 software (AB Sciex). The precursor ions with mass range of 300 – 1500 m/z and calculated charge from +2 to +5 were selected for fragmentation, and MS/MS data was acquired from m/z 60 – 1500 for the three most abundant ions with pulsing mode on for each 2 sec acquisition cycle.

**Data analysis.** Mass spectra analyses were performed by ProteinPilot Software (version 4.0, AB Sciex), using Paragon protein database search algorithm, for generation of peak list, protein identification and quantification. The data analysis parameters were set as follows: sample type, iTRAQ (peptide labeled), Cys Alkylation, MMTS; Digestion: Trypsin; Instrument, QSTAR ESI; Special factors, None; Species, Homo sapien; Quantitate tab, checked; ID focus; Biological modifications; Database Unitprot – Swiss; Thorough ID; Protein Pilot Unused Score of higher than 1.3 (higher than 95% confidence interval). Default precursor and MS/MS tolerance were adopted automatically by the software. The false discovery rate (FDR) of both peptide and protein identification were set to be less than 5%. The sum intensities of reporter ions from various peptides of each protein were automatically calculated by the software based on several peptides of those proteins. The relative intensities of each protein from eight samples in iTRAQ experiments were compared by Mann Whitney U test.

Protein ontology classification was performed using PANTHER classification system (<http://pantherdb.org/>, CA). Accession numbers of proteins were submitted to the protein analysis through evolution relationships categorized system (PANTHER). Proteins were categorized accordingly to related protein classes, biological processes and pathway.

**Western blotting (WB)**. Proteins from the whole plasma samples (un-depleted) were electrophoretically separated on a SDS-PAGE 5-14% gradient gel (Invitrogen) and transferred to PVDF membrane (Millipore) for probing with antibodies at the indication dilutions: anti-AGT (Angiotensinogen; 1:10000, rabbit polyclonal; Abcam) and anti-AT III (Antithrombin-III; 1:10000, rabbit polyclonal; GeneTex Inc). The secondary antibody, HRP Donkey anti-rabbit IgG (1:1000) were purchased from BioLegend. Immunoblotting of a healthy Japanese volunteer without history of Dengue infection was used as an internal control. All immunoblots were performed at least twice. The same dilution of plasma samples was used for Western blots depending on the sensitivity of the specific antibodies (1:120 or 1:360 of plasma dilution). Bands were visualized with enhanced chemiluminescence using Luminata Forte Western HRP substrate (Millipore) and detected by mini-luminescent-image analyzer LAS 4000 system (Fujifilm). Densitometry analysis was performed using Multi Gauge version 3.0 (Fujifilm) and quantifying blots was recorded based on the standard using serial dilutions of the healthy control sample. The relative expression of each band was calculated by the formula that the densitometry value of the test sample is divided by the value of the normal control.
