# Supplementary figures and images for: A Proteomic Approach Identifies Candidate Early Biomarkers to Predict Severe Dengue in Children
Source: PLoS Negl Trop Dis. 2016 Feb 19;10(2):e0004435. doi: 10.1371/journal.pntd.0004435 (PMC4764501; doi:10.1371/journal.pntd.0004435)

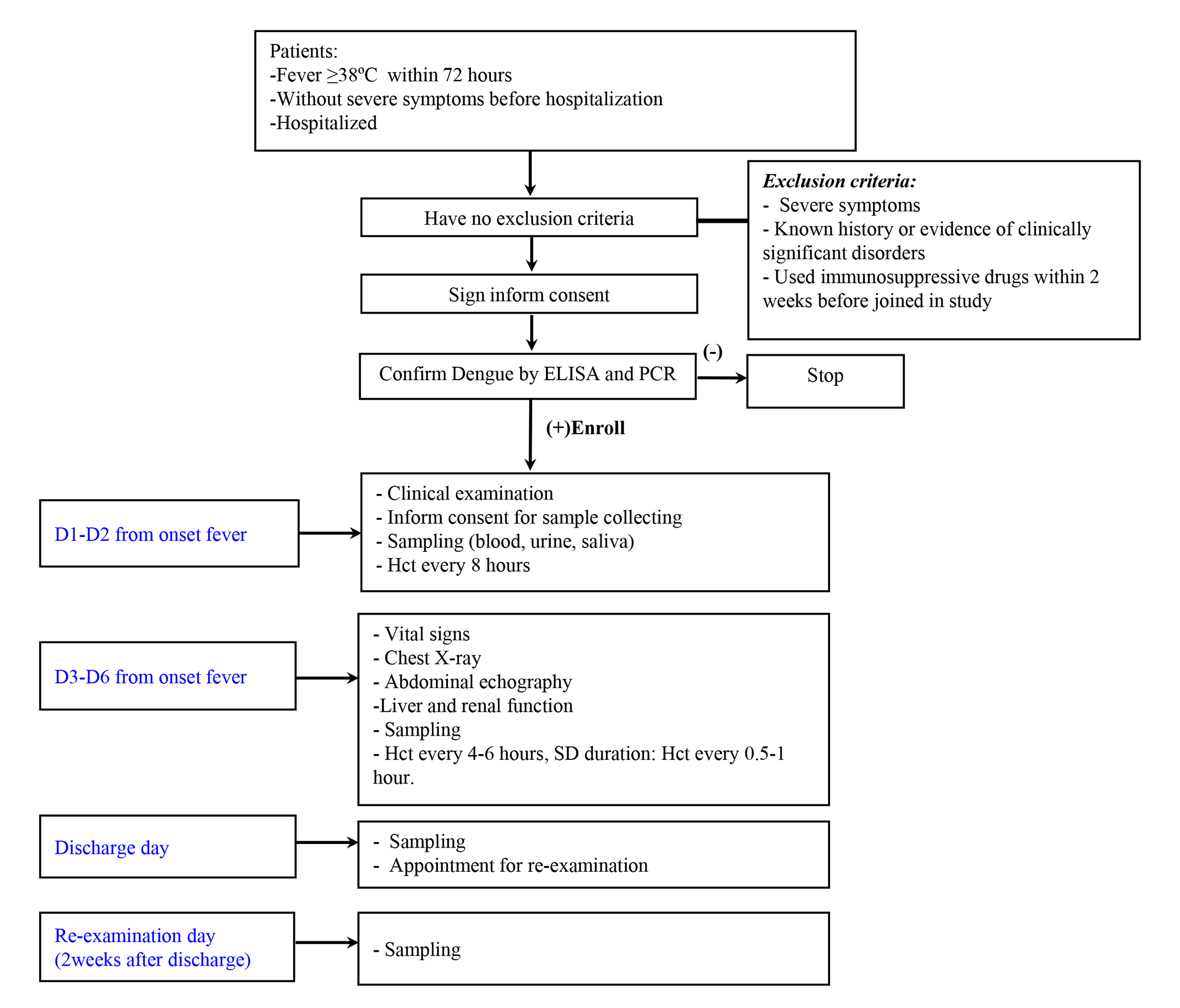

Supplement: S1 Fig — (TIF) [file pntd.0004435.s002.tif]

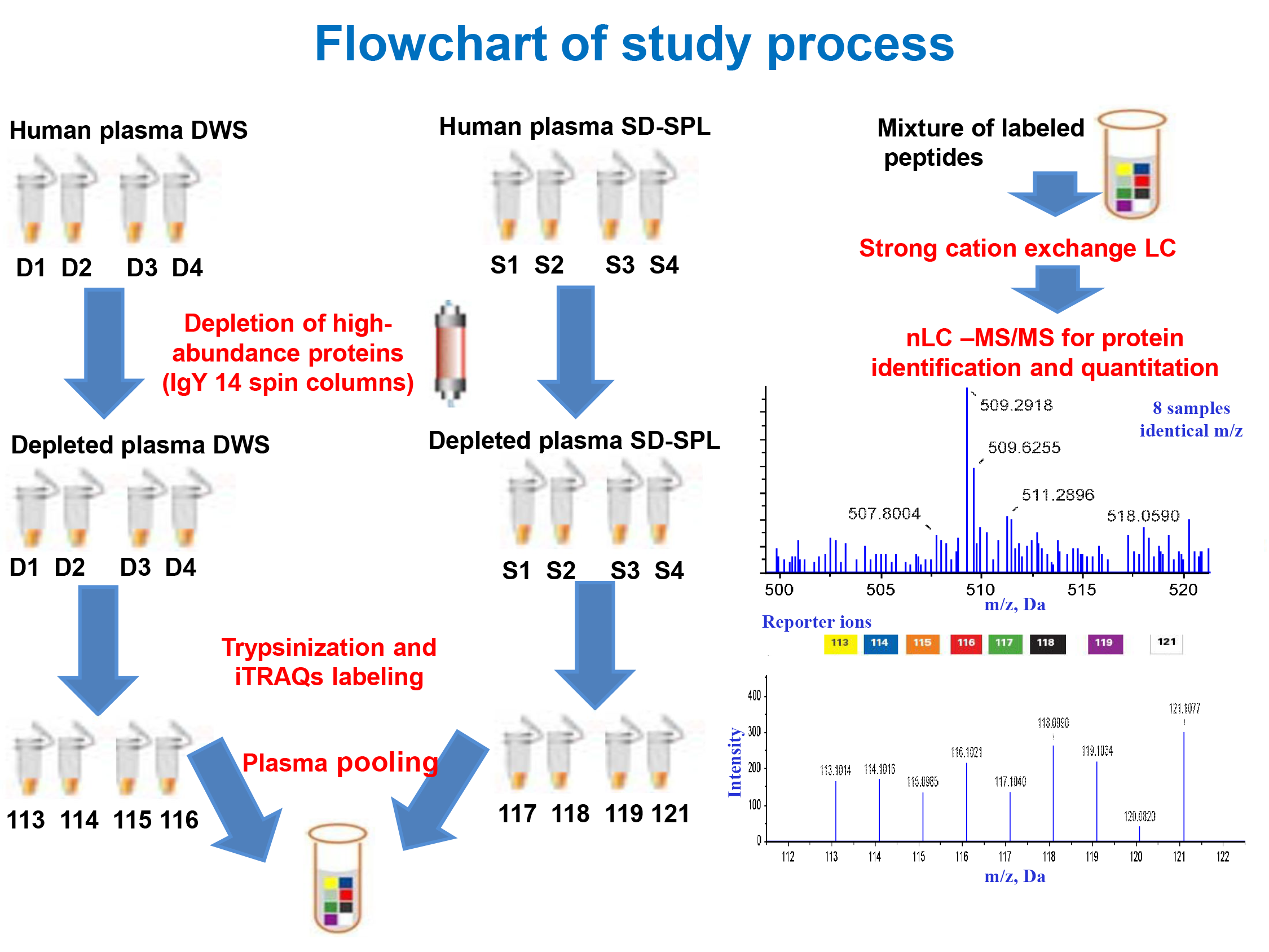

Supplement: S2 Fig — (TIF) [file pntd.0004435.s003.tif]

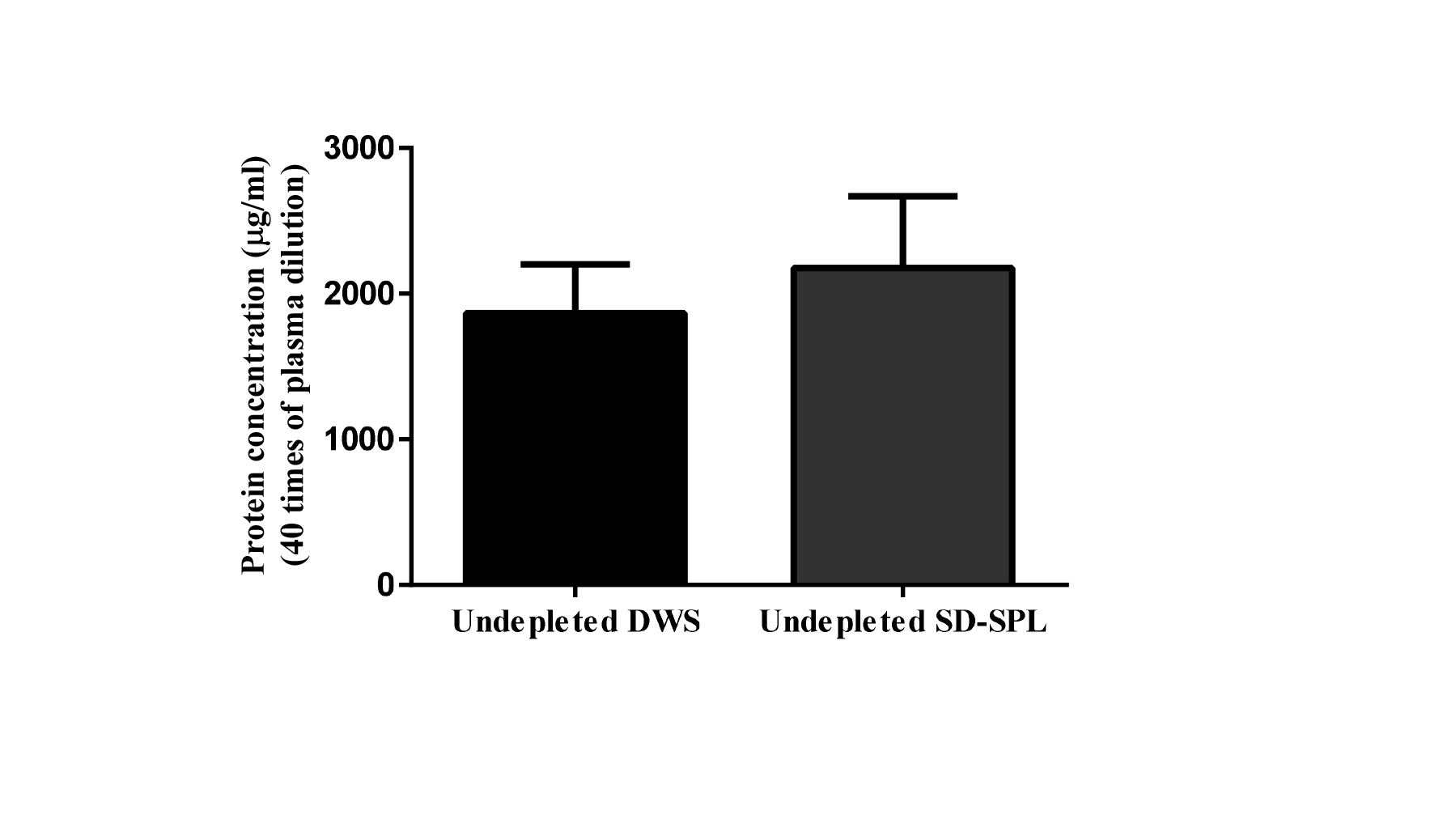

Supplement: S3 Fig — Bar chart represents the median of protein concentration with the upper error bars indicate the 75th percentile of inter-quartile range. (TIF) [file pntd.0004435.s004.tif]
