# Supplementary material for: A Proteomic Approach Identifies Candidate Early Biomarkers to Predict Severe Dengue in Children
Source: PLoS Negl Trop Dis. 2016 Feb 19;10(2):e0004435. doi: 10.1371/journal.pntd.0004435 (PMC4764501; doi:10.1371/journal.pntd.0004435)
Supplement: S1 Table — (DOCX) [file pntd.0004435.s005.docx]

**S1 Table. Classification, sampling time, hematocrit features and fluid management of sixteen patients**

| N | Classification 2009 WHO | % Hct rising (according to age, population*) | % Hct rising (based on Hct min) | Hct min | Isotonic crystalloid solution | |  | Colloid solution | |
| --- | --- | --- | --- | --- | --- | --- | --- | --- | --- |
|  |  |  |  |  | Amount (ml) | Duration (hours) |  | Amount (ml) | Duration (hours) |
| 1 | SD-SPL | 26.76 | 46.56 | 32.00 | 9500 | 10 |  | 0 |  |
| 2 | SD-SPL | 27.03 | 23.36 | 38.10 | 5000 | 25.2 |  | 500 | ND |
| 3 | SD-SPL | 50.00 | 35.00 | 40.00 | 4000 | 17.8 |  | 3600 | 27.5 |
| 4 | SD-SPL | 27.03 | 38.24 | 34.00 | 5000 | 11 |  | 3500 | 38 |
| 5 | SD-SPL | 36.11 | 45.40 | 33.70 | 1000 | 2.8 |  | 3500 | 23.8 |
| 6 | SD-SPL | 22.22 | 15.79 | 38.00 | 3000 | 14 |  | 2000 | 21.7 |
| 7 | DWS | 40.54 | 26.83 | 41.00 | 2500 | 47.5 |  | 0 |  |
| 8 | DWS | 11.11 | 14.29 | 35.00 | 2000 | 21 |  | 0 |  |
| 9 | DWS | 25.00 | 13.64 | 39.60 | 2000 | 22 |  | 0 |  |
| 10 | DWS | 22.22 | 13.99 | 38.60 | 2500 | 25.8 |  | 0 |  |
| 11 | DWS | 4.72 | 12.54 | 33.50 | 0 |  |  | 0 |  |
| 12 | DWS | 61.11 | 18.37 | 49.00 | 500 | ND |  | 0 |  |
| 13 | DWS | 25.83 | 13.25 | 40.00 | 0 |  |  | 0 |  |
| 14 | DWS | 11.67 | 21.45 | 33.10 | 0 |  |  | 0 |  |
| 15 | DWS | 28.61 | 15.75 | 40.00 | 0 |  |  | 0 |  |
| 16 | DWS | 26.67 | 25.62 | 36.30 | 0 |  |  | 0 |  |
| \| *Hct (%) lowest of normal range for Vietnamese: \| \| --- \| \| Male: 41 \| \| Female:37 \| \| 12-16: 37 \| \| 6-11: 36 \| \| 1-5: 34  ND, no data \| | | | | | | | | | |
