# Supplementary material for: A Proteomic Approach Identifies Candidate Early Biomarkers to Predict Severe Dengue in Children
Source: PLoS Negl Trop Dis. 2016 Feb 19;10(2):e0004435. doi: 10.1371/journal.pntd.0004435 (PMC4764501; doi:10.1371/journal.pntd.0004435)
Supplement: S2 Table — (DOCX) [file pntd.0004435.s006.docx]

**S2 Table. Features of SD-SPL patients at the time of shock occurrence**

| Age | Blood sampling (hours before shock) | Temperature  (⁰C) | Pulse rate (b/min) | Blood pressure (mmHg) | Breathing rates/min | Cold extremities | Capillary refill time (seconds) | Mucosal bleeding | Abdomi-nal pain | Enlarged liver | Ascites or pleural effusion | Plasma leakage | Hema-tocrit  (%) | Platelet count  (× 10^3^/μl) | |
| --- | --- | --- | --- | --- | --- | --- | --- | --- | --- | --- | --- | --- | --- | --- | --- |
| 12 | 40 | 36.5 | 85 | 100/80 | 30 | N | >2 | Y | N | N | N | Y | 45 | 30 | |
| 10 | 32 | 36.8 | 110 | 105/80 | 26 | Y | >2 | N | N | N | N | Y | 47 | 46 | |
| 15 | 12 | 37.0 | 100 | 100/80 | 22 | N | >2 | Y | Y | N | N | Y | 47 | 154 | |
| 7 | 41 | 36.5 | 90 | 110/90 | 28 | Y | >2 | N | N | N | N* | Y | 49 | 85 | |
| 10 | 6 | 37.8 | 92 | 80/60 | 28 | N | >2 | N | Y | Y | N | Y | 47 | 28 | |
| 9 | 25 | 36.5 | 120 | 100/80 | 28 | Y | >2 | N | Y | N | N | Y | 44 | 31 | |
| Y, yes  N, no  *After fluid therapy, pleural effusion was observed and treatment of small doses of diuretic was needed | | | | | | | | | | | | | | |  |
