# Supplementary material for: A Proteomic Approach Identifies Candidate Early Biomarkers to Predict Severe Dengue in Children
Source: PLoS Negl Trop Dis. 2016 Feb 19;10(2):e0004435. doi: 10.1371/journal.pntd.0004435 (PMC4764501; doi:10.1371/journal.pntd.0004435)
Supplement: S3 Table — (DOCX) [file pntd.0004435.s007.docx]

**S3 Table. Proteins identified and quantified in iTRAQ experiment**

| N | **Accession** | **Name** | **Cov (%)** | **SD-SPL/DWS** | **p value** |
| --- | --- | --- | --- | --- | --- |
| 1 | sp\|P02768\|ALBU_HUMAN | Serum albumin | 73.6 | 0.7/1.3 | 0.0687 |
| 2 | sp\|P01023\|A2MG_HUMAN | Alpha-2-macroglobulin | 27.7 | 0.8/1 | 0.3175 |
| 3 | sp\|P0C0L4\|CO4A_HUMAN | Complement C4-A | 20.2 | 0.9/1 | 0.1572 |
| 4 | sp\|P0C0L5\|CO4B_HUMAN | Complement C4-B | 20 | 0.8/1 | 0.1572 |
| 5 | sp\|P01871\|IGHM_HUMAN | Ig mu chain C region | 27.4 | 1.1/1 | 0.6644 |
| 6 | sp\|P01857\|IGHG1_HUMAN | Ig gamma-1 chain C region | 44.6 | 1.9/2.1 | 0.6106 |
| 7 | sp\|P01011\|AACT_HUMAN | Alpha-1-antichymotrypsin | 31.2 | 0.9/1 | 0.5337 |
| 8 | sp\|P02790\|HEMO_HUMAN | Hemopexin | 22.3 | 1.1/1 | 0.2347 |
| 9 | sp\|P02774\|VTDB_HUMAN | Vitamin D-binding protein | 20.7 | 1.1/1 | 0.2063 |
| 10 | sp\|P01834\|IGKC_HUMAN | Ig kappa chain C region | 84.9 | 1.8/1.3 | 0.3754 |
| 11 | sp\|P01859\|IGHG2_HUMAN | Ig gamma-2 chain C region | 36.5 | 0.8/1 | 0.7485 |
| 12 | sp\|P04217\|A1BG_HUMAN | Alpha-1B-glycoprotein | 19.1 | 0.9/1 | 0.3361 |
| 13 | sp\|P00738\|HPT_HUMAN | Haptoglobin | 23.4 | 1/1.1 | 0.3361 |
| 14 | sp\|P05155\|IC1_HUMAN | Plasma protease C1 inhibitor | 13.4 | 0.9/1 | 0.5844 |
| 15 | sp\|P01008\|ANT3_HUMAN | Antithrombin III | 23.9 | 1/0.7 | 0.0031 |
| 16 | sp\|P02671\|FIBA_HUMAN | Fibrinogen alpha chain | 17.7 | 1.07/1.06 | 0.9849 |
| 17 | sp\|P00450\|CERU_HUMAN | Ceruloplasmin | 16.8 | 0.9/1.1 | 0.0332 |
| 18 | sp\|Q14624\|ITIH4_HUMAN | Inter-alpha-trypsin inhibitor heavy chain H4 | 7.6 | 1.1/1 | 0.2996 |
| 19 | sp\|P02679\|FIBG_HUMAN | Fibrinogen gamma chain | 7.5 | 1.2/1.1 | 0.895 |
| 20 | sp\|P02675\|FIBB_HUMAN | Fibrinogen beta chain | 8.4 | 1.03/1.05 | 0.8063 |
| 21 | sp\|O60885\|BRD4_HUMAN | Bromodomain-containing protein 4 | 2.1 | 0.7/1 | 0.8617 |
| 22 | sp\|O14529\|CUX2_HUMAN | Homeobox protein cut-like 2 | 3 | 0.96/1 | 0.8356 |
| 23 | sp\|P01009\|A1AT_HUMAN | Alpha-1-antitrypsin | 22.1 | 0.8/1.1 | 0.0861 |
| 24 | sp\|P02765\|FETUA_HUMAN | Alpha-2-HS-glycoprotein | 21 | 0.79/1.01 | 0.0014 |
| 25 | sp\|P0CG06\|LAC3_HUMAN | Ig lambda-3 chain C regions | 41.5 | 1.26/1 | 0.1008 |
|  |  |  |  |  |  |
| 26 | sp\|P00751\|CFAB_HUMAN | Complement factor B | 24.08 | 1.14/1.03 | 0.3175 |
| 27 | sp\|P01019\|ANGT_HUMAN | Angiotensinogen | 12.8 | 1.07/0.9 | 0.0117 |
| 28 | sp\|P02787\|TRFE_HUMAN | Serotransferrin | 15 | 0.93/1.23 | 0.0083 |
| 29 | sp\|P01621\|KV303_HUMAN | Ig kappa chain V-III region NG9 (Fragment) | 33 | 1.5/1.3 | 0.5587 |
| 30 | sp\|Q68DK2\|ZFY26_HUMAN | Zinc finger FYVE domain-containing protein 26 | 11 | 0.73/1.18 | 0.0248 |
| 31 | sp\|P00734\|THRB_HUMAN | Prothrombin | 9.2 | 0.78/1 | 0.0203 |
| 32 | sp\|P68871\|HBB_HUMAN | Hemoglobin subunit beta | 26.5 | 0.98/1 | 0.985 |
| 33 | sp\|P69905\|HBA_HUMAN | Hemoglobin subunit alpha | 16.9 | 2.69/1.13 | 0.5844 |
| 34 | sp\|Q9UFD9\|RIM3A_HUMAN | RIMS-binding protein 3A | 6.9 | 1.95/1.06 | 0.0426 |
| 35 | sp\|Q6IQ23\|PKHA7_HUMAN | Pleckstrin homology domain-containing family A member 7 | 6.2 | 0.98/1.08 | 0.8652 |
| 36 | sp\|Q8WXH0\|SYNE2_HUMAN | Nesprin-2 | 2.1 | 0.8/1.02 | 0.0184 |
| 37 | sp\|Q96L73\|NSD1_HUMAN | Histone-lysine N-methyltransferase, H3 lysine-36 and H4 lysine-20 specific | 3.2 | 1.06/1.05 | 0.9549 |
| 38 | sp\|Q4V328\|GRAP1_HUMAN | GRIP1-associated protein 1 | 3.1 | 0.8/1 | 0.2263 |
| 39 | sp\|P02647\|APOA1_HUMAN | Apolipoprotein A-I | 8.2 | 1.35/1.46 | 0.8063 |
| 40 | sp\|O95255\|MRP6_HUMAN | Multidrug resistance-associated protein 6 | 2 | 0.87/1.0 | 0.0021 |
| 41 | sp\|Q9NUV7\|SPTC3_HUMAN | Serine palmitoyltransferase 3 | 3.7 | 0.43/1 | 0.0078 |
| 42 | sp\|P01861\|IGHG4_HUMAN | Ig gamma-4 chain C region | 27.5 | 1.2/1 | 0.3487 |
| 43 | sp\|P35858\|ALS_HUMAN | Insulin-like growth factor-binding protein complex acid labile subunit | 4.1 | 1.39/1 | 0.22 |
| 44 | sp\|Q9Y4P8\|WIPI2_HUMAN | WD repeat domain phosphoinositide-interacting protein 2 | 8.4 | 1.5/1.01 | < 0.0001 |
| 45 | sp\|Q6ZMZ3\|SYNE3_HUMAN | Nesprin-3 | 3.6 | 0.93/1.04 | 0.1266 |
| 46 | sp\|Q96HE9\|PRR11_HUMAN | Proline-rich protein 11 | 9.4 | 1.89/1.52 | 0.2347 |
| 47 | sp\|Q8TEM1\|PO210_HUMAN | Nuclear pore membrane glycoprotein 210 | 8.5 | 0.42/1 | 0.0135 |
| 48 | sp\|Q8NGK0\|O51G2_HUMAN | Olfactory receptor 51G2 | 7.9 | 0.69/1.03 | 0.001 |
| 49 | sp\|Q7RTS5\|OTOP3_HUMAN | Otopetrin-3 | 8.9 | 0.44/1 | 0.015 |
| 50 | sp\|P04004\|VTNC_HUMAN | Vitronectin | 8.2 | 1.12/1.04 | 0.396 |
| 51 | sp\|O60765\|Z354A_HUMAN | Zinc finger protein 354A | 3.6 | 1.02/1.05 | 0.8652 |
| 52 | sp\|Q2KHM9\|K0753_HUMAN | Uncharacterized protein KIAA0753 | 2.7 | 0.98/1.16 | 0.462 |
| 53 | sp\|Q92771\|DDX12_HUMAN | Putative ATP-dependent RNA helicase DDX12 | 2.1 | 1.22/1.09 | 0.396 |
| 54 | sp\|Q9HBA0\|TRPV4_HUMAN | Transient receptor potential cation channel subfamily V member 4 | 3.1 | 0.92/1.05 | 0.2202 |
| 55 | sp\|Q15582\|BGH3_HUMAN | Transforming growth factor-beta-induced protein ig-h3 | 6.3 | 0.45/1 | 0.0024 |
| 56 | sp\|Q6ZU35\|K1211_HUMAN | Uncharacterized protein KIAA1211 | 2.4 | 0.91/1.09 | 0.462 |
| 57 | sp\|Q8WWN8\|ARAP3_HUMAN | Arf-GAP with Rho-GAP domain, ANK repeat and PH domain-containing protein 3 | 7.4 | 0.72/1 | 0.0248 |
| 58 | sp\|Q96GW7\|PGCB_HUMAN | Brevican core protein | 1.8 | 1.15/1.07 | 0.6106 |
| 59 | sp\|Q9UPQ0\|LIMC1_HUMAN | LIM and calponin homology domains-containing protein 1 | 3.1 | 1/1.01 | 0.6644 |
| 60 | sp\|Q5THJ4\|VP13D_HUMAN | Vacuolar protein sorting-associated protein 13D | 7.3 | 0.71/1.05 | 0.0109 |
| 61 | sp\|P01777\|HV316_HUMAN | Ig heavy chain V-III region TEI | 20.1 | 1.63/1.8 | 0.6373 |
| 62 | sp\|Q9Y2G9\|SBNO2_HUMAN | Protein strawberry notch homolog 2 | 3.1 | 0.82/1.2 | 0.1362 |
| 63 | sp\|Q86VI3\|IQGA3_HUMAN | Ras GTPase-activating-like protein IQGAP3 | 3.2 | 1.2/1.03 | 0.2202 |
| 64 | sp\|P32298\|GRK4_HUMAN | G protein-coupled receptor kinase 4 | 4.9 | 1.22/1.06 | 0.1931 |
| 65 | sp\|Q8N4S9\|MALD2_HUMAN | MARVEL domain-containing protein 2 | 4.8 | 0.86/1.19 | 0.1266 |
| 66 | sp\|Q6FIF0\|ZFAN6_HUMAN | AN1-type zinc finger protein 6 | 3.8 | 0.98/1.29 | 0.1572 |
| 67 | sp\|Q5SZD1\|CF141_HUMAN | Uncharacterized protein C6orf141 | 12.7 | 0.95/1.03 | 0.462 |
| 68 | sp\|A8TX70\|CO6A5_HUMAN | Collagen alpha-5(VI) chain | 2 | 1.16/1.05 | 0.5092 |
| 69 | sp\|Q8WZ42\|TITIN_HUMAN | Titin | 3 | 1.3/1.03 | 0.0363 |
| 70 | sp\|P00747\|PLMN_HUMAN | Plasminogen | 7 | 1.1/1 | 0.4173 |
| Footnote for Supplemental Table 6.  Cov, coverage | | | | | |
